# Nitrogen-doped Carbon with Modulated Surface Chemistry and Porous Structure by a Stepwise Biomass Activation Process towards Enhanced Electrochemical Lithium-Ion Storage

**DOI:** 10.1038/s41598-019-50330-w

**Published:** 2019-10-21

**Authors:** Zhenzhen Nie, Yewei Huang, Beibei Ma, Xiaobin Qiu, Nan Zhang, Xiuqiang Xie, Zhenjun Wu

**Affiliations:** 1 College of Chemistry and Chemical Engineering, Hunan University, Changsha, 410082 P.R. China; 2 College of Materials Science and Engineering, Hunan University, Changsha, 410082 P.R. China

**Keywords:** Energy science and technology, Materials science

## Abstract

Controllable conversion of biomass to value-added carbon materials is attractive towards a wide variety of potential applications. Herein, hydrothermal treatment and KOH activation are successively employed to treat the cheap and abundant camellia oleifera shell as a new carbon raw material. It is shown that this stepwise activation process allows the production of porous nitrogen-doped carbon with optimized surface chemistry and porous structure compared to the counterparts prepared by a single activation procedure. Benefiting from the modulated porous structure, the as-produced porous nitrogen-doped carbon electrode delivered a high reversible capacity of 1080 mAh g^−1^ at a current density of 100 mA g^−1^, which is 3.3 and 5.8 times as high as that of the carbon materials prepared by bare hydrothermal treatment or KOH activation, respectively. Moreover, the optimized surface composition of the porous nitrogen-doped carbon endows it with a highest initial Coulombic efficiency among the three samples, showing great potentials for practical applications. This work is expected to pave a new avenue to upgrade biomass to carbon materials with tunable surface properties and microstructures for target applications.

## Introduction

As one of the most competitive electrochemical energy storage devices, lithium ion batteries (LIBs) have received enormous interest due to their relatively light weight and high energy density^1,2^. Electrode materials play a vital role in meeting the increasing demands on energy density, size, weight, cost, and lifespan of high performance LIBs. Carbon materials are one of the most commercially valued anode materials duo to the low price, low lithium ion intercalation potential and good cycling stability^3^. It is established that heteroatom-doping can induce defects, increase available active sites, and effectively modulate the electronic and chemical properties, thereby enhancing the electrochemical reactivity of carbon materials in LIBs^4,5^. The use of cost-effective biomass has been an attractive facile approach to fabricate carbon materials with appropriate heteroatom-doping, taking advantage of the low cost and composition diversity (such as nitrogen and/or sulfur-containing organic moieties) of biomass precursors^6,7^. In addition, biomass-derived materials usually have the intrinsic hierarchical porous structure and large specific areas, which not only afford favorable electrode/electrolyte interface for charge-transfer reactions, but also facilitate ion transport by offering shorter diffusion pathways to enhance the rate-capability^8^. These features endow biomass-derived carbon materials with satisfactory performances for electrochemical lithium-ion storage.

Diverse plant tissues have been explored as the biomass raw materials to produce porous carbon-based materials due to their natural abundance, such as rice husk^9^, straw^10^, peanut shells^11^, and spongy pomelo peels^12^. Direct pyrolysis under anaerobic environment has been mainly applied to convert the biomass precursor to carbon materials, during which a series of complex, concurrent and consecutive reactions occur before the as-prepared carbon materials are formed at a fixed high temperature^13–15^. In some cases, pore-forming chemicals such as ZnCl_2_ and KOH are often introduced in the pyrolysis process to obtain high surface area and hierarchical porous carbon^16–18^. Technically, biomass-derived carbon materials are typically amorphous and a considerable amount of lithium can be accommodated on edges, surfaces, and nanoscopic cavities of the nanostructures, thereby exhibiting much higher Li-ion storage capacities than graphitic carbon^19^. Consequently, the porous structure significantly affects their specific capacities for electrochemical Li-ion storage^20^. In addition, it should be noted that biomass-derived carbon materials often suffer from low initial Coulombic efficiency (ICE) due to the presence of abundant surface residues trapping Li-ions, such as oxygen-containing groups^21^. In this regard, efforts on modulating the surface properties and microstructures toward enhancing the electrochemical performances (for example, ICE and specific capacity) of the biomass-derived carbon materials is highly desirable but has been lacking in literatures.

As a by-product of camellia oleifera abel, the camellia oleifera shell possesses many advantages, including low cost, rapid regeneration, easy access and environmental friendliness, which favor the mass production of advanced carbon-based materials by the biomass activation from the commercialization prospect. In this paper, we reported a deliberately designed two-step activation process involving hydrothermal treatment and KOH activation to convert the camellia oleifera shell as a new biomass precursor to porous nitrogen-doped carbon with modulated surface chemistry and microstructure. The initial hydrothermal treatment induces several chemical transformation cascades of the biomass component, such as dehydration, condensation, and polymerization, resulting in the formation of pores at the nanoscale. The porous structure facilitates the penetration of molten KOH into the pores driven by capillary forces in the subsequent KOH activation process. This is favorable for the KOH-carbon interaction to finely tune the porous structure. In addition, the surface properties of the final carbon materials are simultaneously optimized. When used as the anode materials for electrochemical Li-ion storage, the as-produced porous nitrogen-doped carbon exhibited superior performances compared to the carbon counterparts prepared by a single activation procedure of either hydrothermal treatment or KOH activation, including improved ICE, significantly increased specific capacity, and enhanced rate-capability.

## Results and Discussions

**Figure 1:**
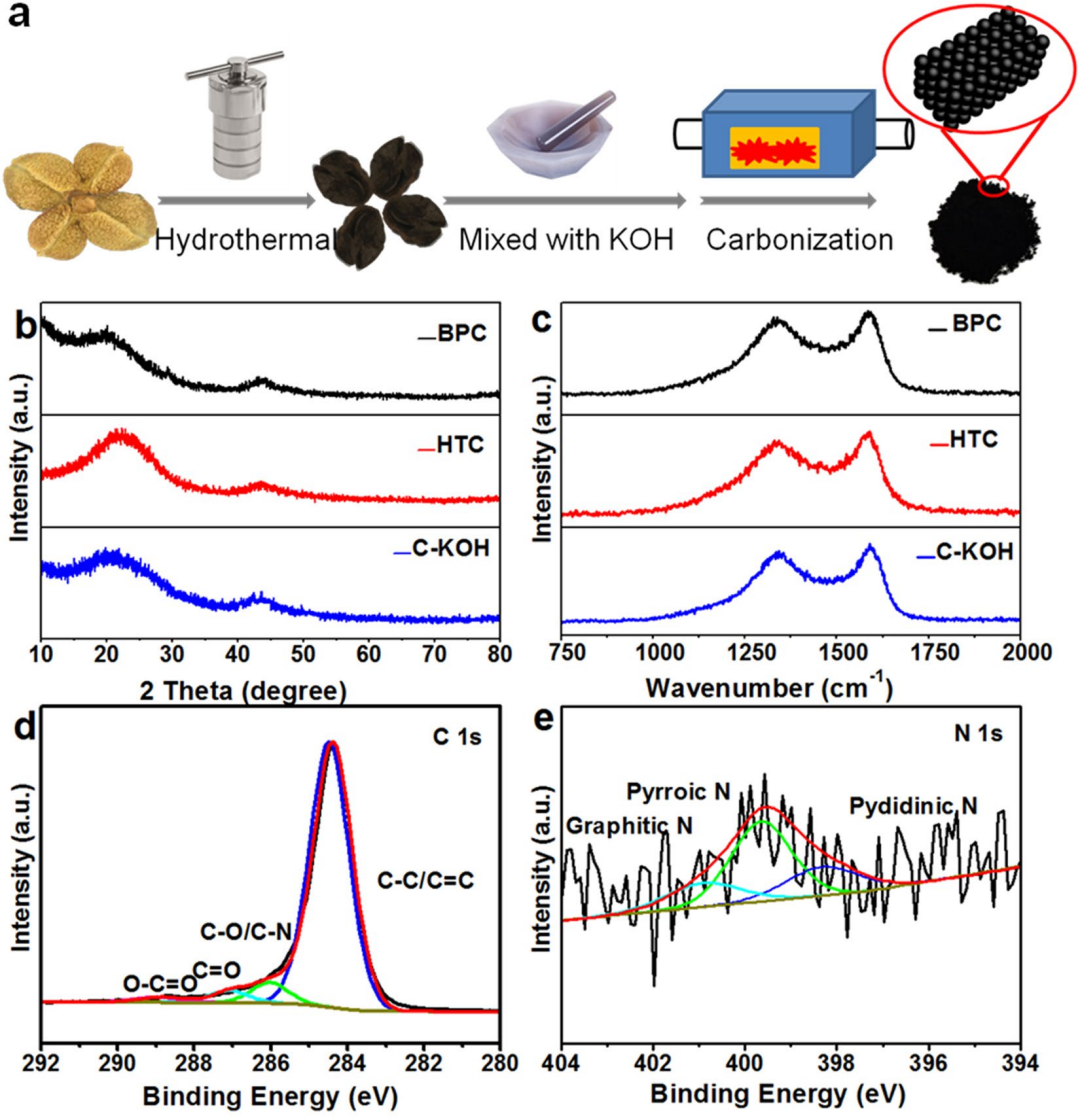
(**a**) Schematic illustration of the formation process of BPC. (**b**) XRD patterns of three samples. (**c**) Raman spectrums of three samples. (**d**) C 1 s XPS spectrum and (**e**) N 1 s XPS spectrum of BPC.

Figure 1a schematically illustrates the conversion of camellia oleifera shells to biomass-derived porous carbon (BPC) by the deliberately designed stepwise protocol. Briefly, camellia oleifera shells were thoroughly washed and subjected to hydrothermal treatment first. The obtained product was then mixed with KOH and sintered at high temperature. For comparison, bare hydrothermal treatment or KOH activation was employed to treat the camellia oleifera shell, and the products are denoted as HTC and C-KOH, respectively. X-ray diffraction (XRD) patterns of the three samples are presented in Fig. 1b. Two broad characteristic peaks are located at around 23° and 43°, which correspond to the (002) and (100) plane of carbon, respectively, suggesting that the three samples are disordered or amorphous^22^. Raman has been widely applied to characterize carbon materials, which is sensitive to the slight structural changes. Raman spectra of BPC, HTC, C-KOH are displayed in Fig. 1c. The three samples have D-band and G-band located at around 1338 cm^−1^ and 1584 cm^−1^, corresponding to the defect/disorder-induced structures in the graphene layers of carbon materials, and the vibration of sp2-bonded carbon atoms in a two-dimensional hexagonal lattice, respectively^23^. The relative strength intensity (I_D_/I_G_) represents the degree of defect in the carbon material and a higher value stands for more defects^24,25^. The values of I_D_/I_G_ for BPC, HTC, and C-KOH are comparable, which are 0.92, 0.90, and 0.91, respectively.

X-ray photoelectron spectroscopy (XPS) measurements have been conducted to investigate the chemical compositions and surface properties of the carbon materials. In the survey spectrum of BPC (Fig. S1a), there are two obvious peaks appearing at 284.7 and 532.5 eV, correspond to C 1 s and O 1 s, respectively. Similar XPS survey spectrum has been found for HTC and C-KOH (Fig. S1b,c). As shown in Fig. 1d, the high-resolution C 1 s spectrum of BPC shows four peaks at 284.3, 286.0, 287.8 and 289.0 eV, corresponding to C-C/C=C, C-O/C-N, C=O, and O-C=O, respectively^26^. Besides, as can be seen from Fig. 1e, the high resolution of N 1 s can be deconvoluted to three peaks located at 400.9, 399.6, and 398.2 eV, which are assigned to graphitic N, pyrrolic N, and pyridinic N, respectively^27^. The nitrogen-dopants originate from the organic nitrogen-containing components in the biomass precursor and are beneficial to lithium binding in different manners, either by the neighboring carbon of graphitic N or interacting with pyridinic N atoms^28,29^. Based on the XPS result, the carbon, oxygen, and nitrogen contents in the BPC are 94.74 at%, 4.96 at%, and 0.3 at%, respectively. As displayed in Fig. S2, carbon, nitrogen and oxygen are homogeneously distributed in BPC. Based on the XPS results of HTC shown in Fig. S3, the carbon, oxygen, and nitrogen contents are calculated to be 92.76 at%, 6.15 at%, and 1.09 at%, respectively. While the carbon, oxygen and nitrogen contents in C-KOH are 89.27 at%, 9.54 at%, and 1.19 at%, respectively (Fig. S4). In comparison, BPC has the minimum oxygen content, which reveals that the successive hydrothermal treatment and KOH activation decrease the oxygen content in the carbon materials compared to the single procedure. The decreased oxygen functional groups can increase the electrical conductivity of carbon materials, as has been confirmed in the previous literature, which is favorable for the rate performance for electrochemical Li-ion storage^30^.

**Figure 2:**
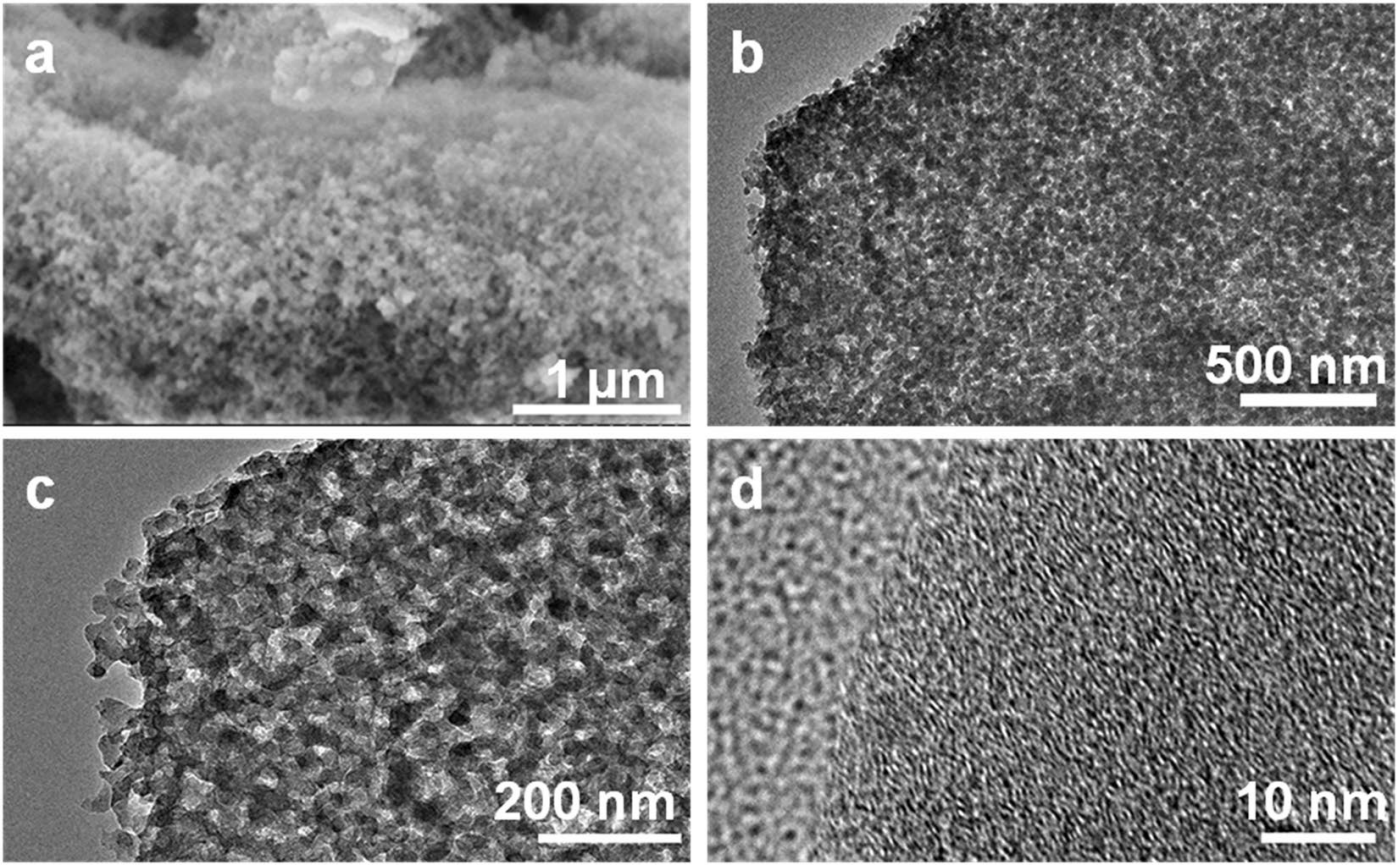
(**a**) SEM, and (**b**–**d**) TEM images of BPC with different magnifications.

The morphologies of the obtained carbon materials have been investigated by scanning electron microscopy (SEM) and transmission electron microscopy (TEM). As can be seen from Fig. S5a, HTC features porous structure with nanoparticles interconnected with each other. The porous structure has been further confirmed by the TEM image (Fig. S5b**)**. This suggests that the hydrothermal treatment of the camellia oleifera shell effectively induces reactions of the organic components in the biomass precursor, such as dehydration, condensation, and polymerization, thereby generating porosity in the as-prepared carbon materials. This induces an important morphological transformation of the bulk biomass precursor, which facilitates the carbon-KOH interactions in the following KOH activation process. As shown in Fig. 2a, BPC prepared by the successive hydrothermal treatment and KOH activation inherits the porous structure of HTC, but the smaller nanoparticles, which can be ascribed to the etching effect of KOH on carbon materials at elevated temperatures^31^. The microstructure of BPC has been further investigated by TEM. From the Fig. 2b it can be discovered that the BPC features abundant pores formed by interconnected particles, which agrees well with the SEM result. The magnified TEM image shown in Fig. 2c further reveals the details of the morphology of the BPC material. Many nanoscale pores were observed, forming a three-dimensional (3D) connected porous structure. The 3D architecture not only maximizes electrode/electrolytes interface for charge transfer reaction, but also favors lithium-ion transport by shortening the diffusion pathways^32^. High-resolution transmission electron microscopy (HRTEM) image clearly shows that there are more large quantities of micropores and channels in BPC (Fig. 2d) than that of HTC (Fig. S5c) and C-KOH (Fig. S6c). These pores are favorable for the Li-ion storage to enhance the capacity^33^. In contrast, C-KOH prepared by the traditional one-step KOH-activation method mainly comprises large and smooth bulk with small amounts of pores (Fig. S6a,b), which unambiguously reveals that the initial hydrothermal treatment can effectively promote the carbon-KOH interactions to modulate the formation of the porous structure.

**Figure 3:**
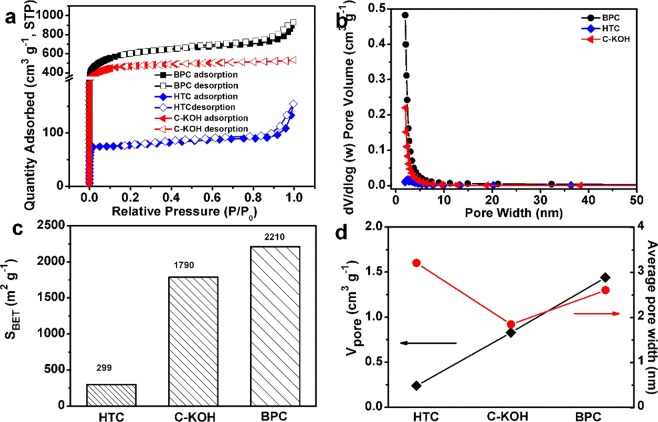
(**a**) Nitrogen adsorption-desorption isotherms. (**b**) Pore size distributions. (**c**) Pore volumes and average pore widths, and (**d**) BET surface areas of HTC, C-KOH, and BPC.

The porosity of the obtained materials has been investigated by N_2_ adsorption-desorption experiments. Figure 3a shows the he N_2_ adsorption-desorption isotherms of the three samples. In particular, BPC has an obviously upward trend at the low relative pressure (P/P_0_ < 0.1), corresponding to the absorption of N_2_ in the micropores. The obvious hysteresis loop (0.5 < P/P_0_ < 1.0) and a sharp rise in the N_2_ isotherm at relatively high pressure from 0.85 to 1.0 (P/P_0_) suggest the existence of mesopores and macropores^34^. The small amount of macropores may derive from the aggregation of carbon particles. For HTC, the hysteresis loop appears at 0.3 < P/P_0_ < 1.0 and has a sharp rise at 0.8 < P/P_0_ < 1.0. While there is no obvious hysteresis loop C-KOH. The pore size distributions for three samples were estimated by the density functional theory (DFT) and shown in Fig. 3b. It is clear that the three samples are mainly composed of micropores and mesopores between 1 and 5 nm. The contribution of micropore (<2 nm) volume to the total pore volume (V_µ_/V_T_) was determined from Kelvin equation taking a relative pressure limit (P/P_0_) of 0.15. As shown in Fig. S7, BPC displays an obvious increase of micropores compared to HTC, which can be ascribed to the rapid volatilization of light organics and amorphization of carbonaceous segments at a relatively high temperature. In addition, a higher portion of micropores of BPC compared to C-KOH can be observed, suggesting that KOH-activation can effective modulate the microporous structure by reacting chemically with carbon^35^. As shown in Fig. 3c, the average pore widths of three samples follow the order of HTC > BPC > C-KOH and the pore volumes of BPC, HTC, C-KOH are 1.44, 0.24, 0.83 cm^3^ g^−1^, respectively. As summarized in Fig. 3d, the Brunauer-Emmett-Teller (BET) specific surface areas of HTC and C-KOH are 299 m^2^ g^−1^ and 1790 m^2^ g^−1^, respectively. BPC has the highest surface area of 2210 m^2^ g^−1^ among the three samples, indicating that hydrothermal treatment and KOH activation cooperatively modulate the mesopores and micropores and greatly increase the specific surface area^36,37^. The higher specific surface area of BPC could effectively enhance electrochemical active sites for the storage of lithium ion, resulting in enhanced lithium storage and adsorption capability^38^.

**Figure 4:**
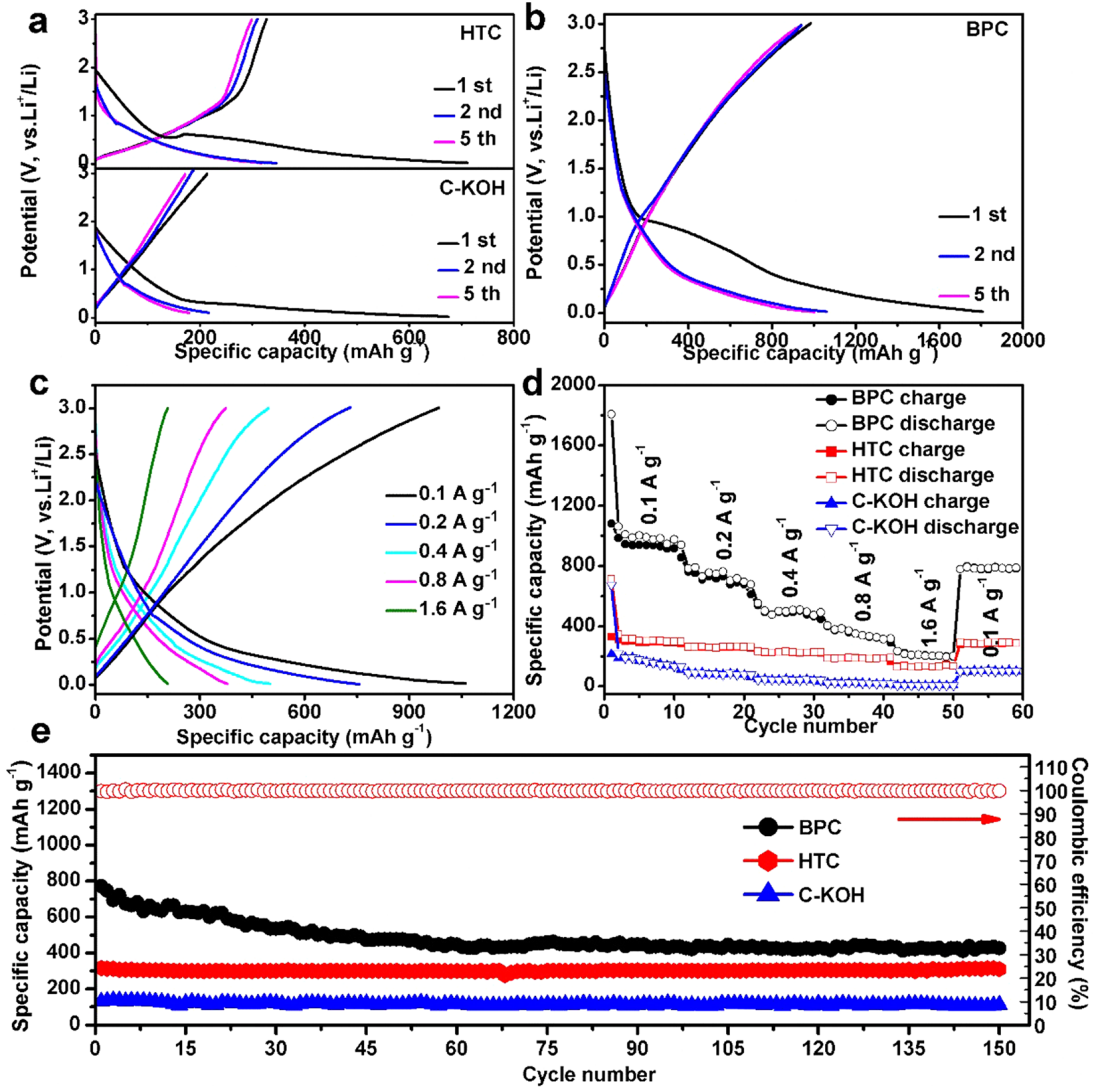
Galvanostatic charge and discharge profiles of HTC, C-KOH (**a**) and BPC (**b**) at 100 mA g^−1^ from 0.01 V to 3.00 V for the 1st, 2nd and 5th cycle. (**c**) Charge-discharge curves of BPC at various current densities. (**d**) Cycling performances of BPC at different current densities from 0.1 A g^−1^ to 1.6 A g^−1^. (**e**) Cycling capability of three samples at a current density of 0.2 A g^−1^.

The electrochemical performances of the as-prepared carbon materials for lithium-ion storage have been tested by assembling coin type cells (2032 type). Figure 4a,b show the discharge/charge curves of three carbon material electrodes in the voltage range of 0.01–3.00 V at a current density of 100 mA g^−1^ at room temperature. As shown in Fig. 4a, the initial reversible specific capacities of HTC and C-KOH is 328 mAh g^−1^ and 187 mAh g^−1^, respectively. As shown in Fig. 4b, the initial lithiation and delithiation capacities of BPC are 1808 and 1080 mAh g^−1^, respectively. The initial reversible capacity of BPC is 3.3 and 5.8 times as high as that of HTC and C-KOH, respectively. It is worth noting that the first reversible capacity of BPC is 2.9 times as high as the theoretical capacity of graphite (372 mAh g^−1^), indicating that there may be other lithium storage scheme existing in BPC in addition to the conventional graphite intercalation mechanism. It is proposed that BPC composed of nano-sized pores provides a large number of lithium adsorption/desorption sites on edges, surfaces, and nanoscopic cavities of the nanostructures, thereby delivering high lithium-ion storage capacities^39^. The BPC electrode has an initial Coulombic efficiency (ICE) of 59.7%. The irreversible capacity in the first cycle can be attributed to the formation of solid electrolyte interface (SEI) layer or the irreversible trapping of lithium by the residual [H] and [O] atoms on the surface of carbon material^40^. Such capacity loss in the initial cycles is common for biomass-derived carbon materials^3,41,42^. Notably, the ICE of BPC is higher than that of HTC (46.1%), C-KOH (27.8%), and most biomass-derived carbon materials, such as Ox horn (59.5%)^40^, Coir pith (44%)^30^ and Hazelnut shells (45.64%)^37^. This suggests the stepwise treatment modulates the surface properties of the carbon materials simultaneously, which is favorable for enhancing the reversible lithium-ion storage. In the second cycle, a high capacity of 1061 mAh g^−1^ has been achieved. After 5 cycles, the reversible capacity can be obtained at 1001 mAh g^−1^ and the Coulombic efficiency stabilizes at around 97.1%. Figure 4c displays the galvanostatic charge-discharge profiles of BPC at different current densities ranging from 100 mA g^−1^ to 1.6 A g^−1^. It can be seen that the charge/discharge curves still maintain a kinetic feature at very high current densities in comparison to that at low current densities, revealing a facile charge transport process. The rate capabilities of the three materials were compared in Fig. 4d. The cells were first cycled at a current density of 0.1 A g^−1^ for 10 cycles, and then at various current densities from 0.2 A g^−1^ to 1.6 A g^−1^ each for 10 cycles. As for BPC, desirable reversible capacities of 1808, 783, 553, and 379 mAh g^−1^ have been achieved at the current density of 0.1, 0.2, 0.4, and 0.8 A g^−1^, respectively. BPC electrode exhibits a promising rate-performance. Even at a high current density of 1.6 A g^−1^, a high reversible capacity of 236 mAh g^−1^ has been obtained. In contrast, at the current density of 1.6 A g^−1^, HTC and C-KOH only deliver reversible capacities of 128 and 6.2 mAh g^−1^, respectively. When the current density turns back to 0.1 A g^−1^, the specific capacity reaches ~790 mAh g^−1^. Figure S8 compares the Nyquist plots of these three samples. The Nyquist plots are typically composed of a depressed semicircle in the moderate frequency region and a line in the low frequency region. It can be seen that the semicircle of BPC is smaller than that of the HTC and C-KOH materials, indicating that the BPC possesses higher charge transfer efficiency. In combination with the N_2_ adsorption-desorption results, it can be concluded that the predominant rate capability of BPC can be ascribed to: (i) the three-dimensional porous structure that favors the rapid transport of Li^+^ on the surface and within the electrode^43^, and (ii) the high charge transfer efficiency for electrochemical redox reactions. The BPC electrode also has a good cycle stability. As shown in Fig. 4e, the reversible capacities of BPC electrode stabilize at 430 mAh g^−1^ after 150 cycles at a current density of 0.2 A g^−1^. In contrast, HTC and C-KOH without microstructure modulation only deliver reversible capacities of 311 and 113 mAh g^−1^, respectively.

Figure S9 shows the cyclic voltammograms (CV) curves of BPC electrodes at a scan rate of 0.1 mV s^−1^. In the first cycle, the reduction peak at around 0.75 V is ascribed to the formation of SEI on the electrode surface. The CV curves almost overlapped in the following cycles, suggesting the formation of a stable SEI layer. The redox current above 0.5 V is ascribed to the faradic capacitance on the surface/edge sites of the carbon product, while the current in the range of 0.01–0.5 V is attributed to the intercalation of Li^+^ ^44^.

**Figure 5:**
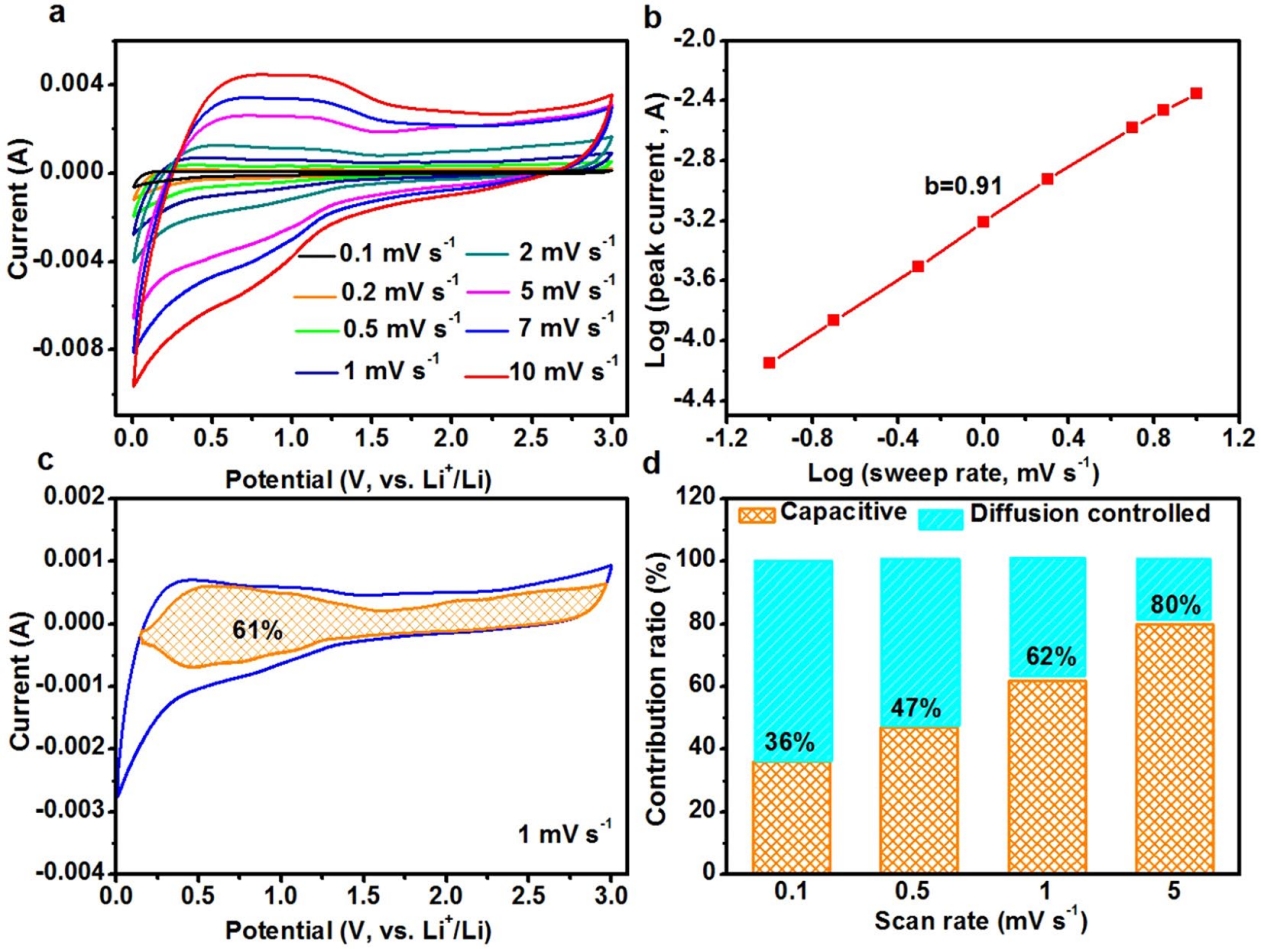
(**a**) CV curves of the BPC electrode at various scan rates from 0.1 to 10 mV s^−1^. (**b**) Determination of the b value using the relationship between peak current and scan rate. The redox peak at 0.7 V was used for the calculations. (**c**) Separation of the capacitive and diffusion currents at a scan rate of 1 mV s^−1^. (**d**) Contribution ratio of the capacitive and diffusion-controlled charge versus scan rate.

Sweep-rate-dependent CV experiments have been performed to investigate the kinetics and charge storage mechanism of the BPC electrode. The CV curves of the BPC electrode at different scan rates is shown in Fig. 5a. The broad cathodic and anodic peaks are equivalent to the galvanostatic charge-discharge profiles. With a 100-times increase in scan rate from 0.1 to 10 mV s^−1^, the broad peaks are maintained and not significantly altered in all CV curves. In ordinary cases, the current (*i*) and sweep rates (*v*) comply with the following power law^45^.

1 $$i=a{v}^{b}$$

Where *a* and *b* are adjustable values. In particular, the *b*-value of 0.5 indicates a totally diffusion-controlled process, whereas 1.0 indicates a capacitive process. The *b*-value can be determined by the slope of the log(*v*) against log(*i*) plots^46^. As shown in Fig. 5b, the *b* value for the BPC anode is 0.91, suggesting that the majority charge process shows capacitive characteristics^47^. The total capacitive contribution to the current response could be further quantitatively determined by separating the current response (*i*) at a specific potential (*v*) into capacitive effects (*k*_1_*v*) and diffusion-controlled processes (*k*_2_*v*^1/2^) according to^48^

2 $$i(V)={k}_{1}v+{k}_{2}{v}^{1/2}$$

Where *k*_1_ and *k*_2_ are constants at a particular sweep rate. We have used this methodology to determine the fraction of the total stored charge from capacitive processes. For BPC electrode, around 61% of the total current (and therefore, the capacity) is capacitive at 1 mVs^−1^, which is denoted by the orange shaded region in Fig. 5c. In addition, the capacitive contribution gradually improves with the increase of scan rate and achieves a maximum value of 80% at 5 mV s^−1^ (Fig. 5d). A comparison of the fraction of the total stored charge from capacitive processes as a function of sweep rate for HTC and C-KOH are shown in Figs S10 and S11 respectively. The capacitive contribution for HTC and C-KOH are significantly lower than that for the BPC. At a scan rate of 5 mV s^−1^, the capacitive contribution is as high as 80% for BPC electrode, which is much higher than that of HTC (17%) and C-KOH (37%). On the other hand, it can be seen that the diffusion controlled process, *i*.*e*., Li^+^ intercalation into carbon layers, contributes predominantly to the charge storage for the HTC and C-KOH electrodes. Based on the XPS results of HTC shown in Fig. S3, the carbon content is calculated to be 92.76 at%. While the carbon contents in C-KOH is reduced to 89.27 at% (Fig. S3). Consequently, C-KOH exhibits inferior lithium storage performance compared to HTC.

The kinetic analysis indicates that the predominant charge storage over BPC originates from the capacitive process. Consequently, it is reasonable to conclude that the superior Li-ion storage capacity of BPC compared to that of HTC and C-KOH is mainly ascribed to its high specific surface area and large pore volume, which is realized by the rationally designed protocol to convert the biomass precursor to carbon materials. Specifically, the initial hydrothermal treatment induces reactions of the organic components in the biomass precursor, such as dehydration, condensation, and polymerization, thereby effectively introducing the mesoporous structure. Since KOH has a relatively low melting point of 380 °C, the mesoporous structure allows the penetration of molten KOH into the pores driven by capillary forces at elevated temperatures, thereby facilitating the carbon-KOH interactions in the following KOH activation process. This affords to finely tune the microporous structure and produce BPC with the highest specific surface areas. Since additional Li-ions can be accommodated on edges, surfaces, and nanoscopic cavities, the amorphous BPC with the highest specific surface areas and largest pore volume delivers the highest specific Li-ion storage capacity than that of HTC and C-KOH. In addition, the large surface area also can supply a sufficient contact area between electrode and electrolyte favor the fast Li-ion diffusion, which benefits the high-rate charging/discharging.

## Conclusion

In summary, hierarchical porous nitrogen-doped carbon materials have been successfully prepared from the cheap camellia oleifera shell as a new biomass precursor through a stepwise hydrothermal treatment and KOH activation process. The as-prepared carbon materials exhibit a high specific surface area of 2210 m^2^ g^−1^ and a pore volume of 1.44 cm^3^ g^−1^, both of which exceed that of the counterparts prepared by bare hydrothermal treatment or KOH activation. In addition, the surface oxygen-containing groups are simultaneously reduced. When used as anode materials for Li-ion storage, the as-prepared porous nitrogen-doped carbon material exhibited an improved ICE, a high reversible capacity of about 1080 mAh g^−1^ at a current density of 100 mA g^−1^, and excellent rate-capability, outperforming the carbon counterparts prepared by bare hydrothermal treatment or KOH activation. The product also exhibited a good stability against cycling and a high reversible capacity of 430 mAh g^−1^ after 150 cycles at a current density of 200 mA g^−1^. These results indicate that the combination of hydrothermal treatment and KOH activation is an effective protocol to modulate the microstructure and surface properties of biomass-derived carbon, which could also be extended to prepare value-added carbon materials by biomass conversion towards applications in fuel cells, adsorbents and catalysis.

## Methods

### Materials preparation

All chemicals used in this work were the analytical grade. An ultra-pure purification system (ECO-S15Q, Hitech Instruments Co. Ltd., Shanghai, China) was used to produce 18.2 MΩ/cm water in all experiments. Camellia oleifera shell was produced in Hunan Province, China. Firstly, the camellia oleifera shells were washed with DI water several times and dried at 80 °C for 12 h. Secondly, the dried camellia oleifera shells were subjected to hydrothermal treatment at 180 °C for 12 h and then filtrated. Thirdly, the product was freeze-dried and carbonized in a tube furnace under nitrogen atmosphere at 800 °C for 2 h. Afterwards, the carbonized camellia olfera shells were mixed with KOH at a KOH/C mass ratio of 4:1 and sintered at 800 °C for 2 h under nitrogen atmosphere. Finally, the as-obtained product was washed with dilute hydrochloric acid (1 M) and deionized water until the pH reached around 7, and then dried in an oven at 80 °C for 12 h. This biomass-derived porous carbon is denoted as BPC. For comparison, bare hydrothermal treatment or KOH activation was employed to treat the camellia oleifera shell, and the products are denoted as HTC and C-KOH, respectively.

### General characterization

X-ray diffraction (XRD) measurements were taken with X-ray diffraction (Bruker D8 Advance diffractometer, Cu Kα1). The Brunauer-Emmett-Teller specific surface areas, total pore volume and average pore diameter were recorded on a Quantachrom NOVA1000e system by N_2_ adsorption-desorption isotherms at 77 K. The Raman spectra were carried out on Renishaw Invia. The morphologies of all products were investigated by scanning electron microscopy (SEM) on Hitachi, S-4800. And transmission electron microscopy (TEM) and high-resolution transmission electron microscopy (HRTEM) were conducted on Hitachi, JEM-2010. The X-ray photoelectron spectroscopy (XPS) was conducted on Thermo Fisher Scientific, K-Alpha 1063. Binding energies of all XPS spectra were calibrated by the C 1 s binding energy of adventitious carbon contamination which was taken to be 284.8 eV.

### Electrochemical measurements

The electrochemical performances of the BPC and the counterparts were evaluated by assembling coin cells (2032 type). To prepare working electrodes active materials, carbon black and polyvinylidene fluoride (PVDF) binder at a weight ratio of 70:20:10 were mixed with N-methyl-2-pyrrolidinone (NMP). The slurry was pasted on a Cu foil and dried at 60 °C for 12 h in vacuum oven. The loading mass of active materials is around 1.4 mg, corresponding to an areal mass loading of 1.8 mg cm^−2^. The obtained electrodes were assembled in an argon-filled glove box using lithium metal as the counter electrode and Celgard 2400 as the separator. The electrolyte was 1.0 mol L^−1^ LiPF_6_ in ethylene carbonate/dimethyl carbonate (the volume ratio is 1:1). The galvanostatic charge-discharge tests were measured on LAND CT-2001A at 25 °C in the voltage range of 0.01–3.00 V at different current densities. The cyclic voltammograms (CV) were tested at scanning rates from 0.1 mV s^−1^ to 10 mV s^−1^ between 0.01 and 3.0 V on CHI 660E. For the electrochemical impedance spectroscopy (EIS), the excitation amplitude applied to the cells was 5 mV and the frequency range is 0.01–100 kHz.

## Supplementary information

Supplementary information
