# Supplementary material for: Nitrogen-doped Carbon with Modulated Surface Chemistry and Porous Structure by a Stepwise Biomass Activation Process towards Enhanced Electrochemical Lithium-Ion Storage
Source: Sci Rep. 2019 Oct 21;9:15032. doi: 10.1038/s41598-019-50330-w (PMC6803759; doi:10.1038/s41598-019-50330-w)
Supplement: Supplementary file 1 — Supplementary information [file 41598_2019_50330_MOESM1_ESM.docx]

***Supplementary information for the manuscript***

**Nitrogen-doped Carbon with Modulated Surface Chemistry and Porous Structure by a Stepwise Biomass Activation Process towards Enhanced Electrochemical Lithium-Ion Storage**

Zhenzhen Nie*^a,†^*, Beibei Ma*^a,†^*, Xiaobin Qiu*^a^*, Yewei Huang*^a^*, Nan Zhang*^b*^*, Xiuqiang Xie*^b*^*, Zhenjun Wu*^a*^*

*^a^* *College of Chemistry and Chemical Engineering, Hunan University, Changsha 410082, P. R. China. E-mail:* [*wooawt@163.com*](mailto:wooawt@163.com)

*^b^ College of Materials Science and Engineering, Hunan University, Changsha 410082, P. R. China. E-mail: nanzhang@hnu.edu.cn;* [*xiuqiang_xie@hnu.edu.cn*](mailto:xiuqiang_xie@hnu.edu.cn)

**Contents list**

**Fig. S1** The survey spectrum of BPC (a), HTC (b), C-KOH (c).

**Fig. S2** Elemental mappings of BPC. (a) Carbon, (b) nitrogen, and (c) oxygen.

**Fig. S3** (a) C 1s, (b) N 1s, and (c) O 1s XPS spectrum of HTC.

**Fig. S4** (a) C 1s, (b) N 1s, and (c) O1s XPS spectrum of C-KOH.

**Fig. S5** (a) SEM, (b) TEM, and (c) HRTEM images of HTC.

**Fig. S6** (a) SEM, (b) TEM, and (c) HRTEM images of C-KOH.

**Fig. S7** Contribution of micropore volume to the total pore volume for HTC, C-KOH, and BPC.

**Fig. S8** Nyquist plots of BPC, HTC, and C-KOH.

**Fig. S9** First five cycles CV curves of the BPC sample at a scan rate of 0.1 mV s^-1^.

**Fig. S10** (a) CV curves of the HTC electrode at various scan rates from 0.1 to 10 mV s^-1^. (b) Determination of the b value using the relationship between peak current and scan rate. The redox peak at 0.7 V was used for the calculations. (c) Separation of the capacitive and diffusion currents at a scan rate of 1 mV s^-1^. (d) Contribution ratio of the capacitive and diffusion-controlled charge versus scan rate.

**Fig. S11** (a) CV curves of the C-KOH electrode at various scan rates from 0.1 to 10 mV s^-1^. (b) Determination of the b value using the relationship between peak current and scan rate. The redox peak at 0.7 V was used for the calculations. (c) Separation of the capacitive and diffusion currents at a scan rate of 1 mV s^-1^. (d) Contribution ratio of the capacitive and diffusion-controlled charge versus scan rate.


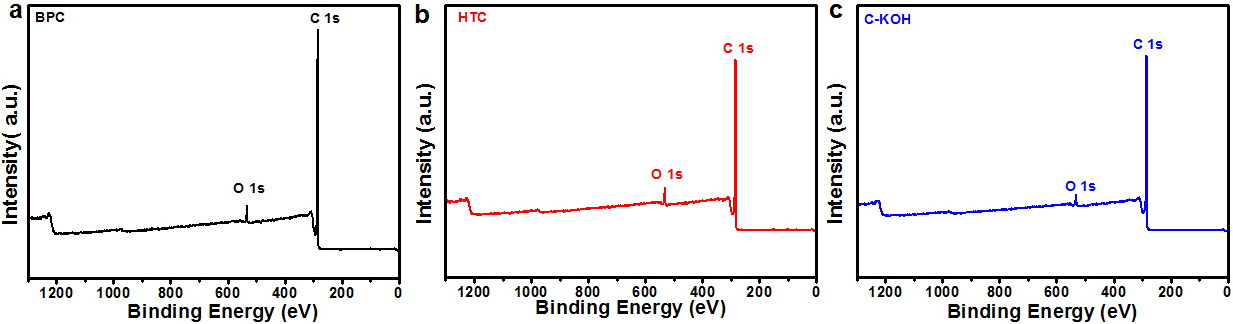


**Fig. S1** The survey spectrum of BPC (a), HTC (b), and C-KOH (c).


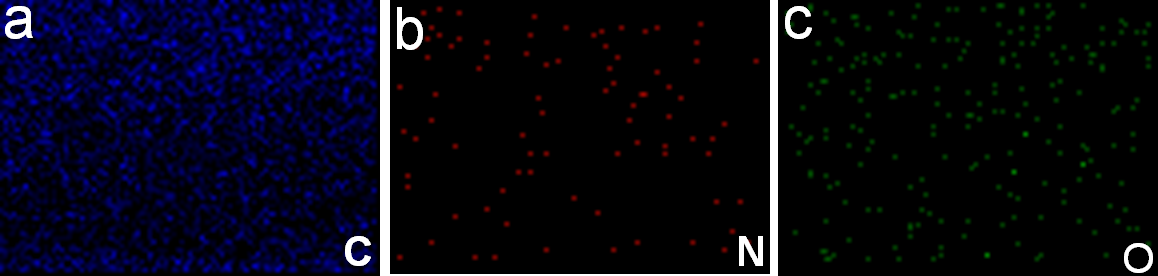


**Fig. S2** Elemental mappings of BPC. (a) Carbon, (b) nitrogen, and (c) oxygen.


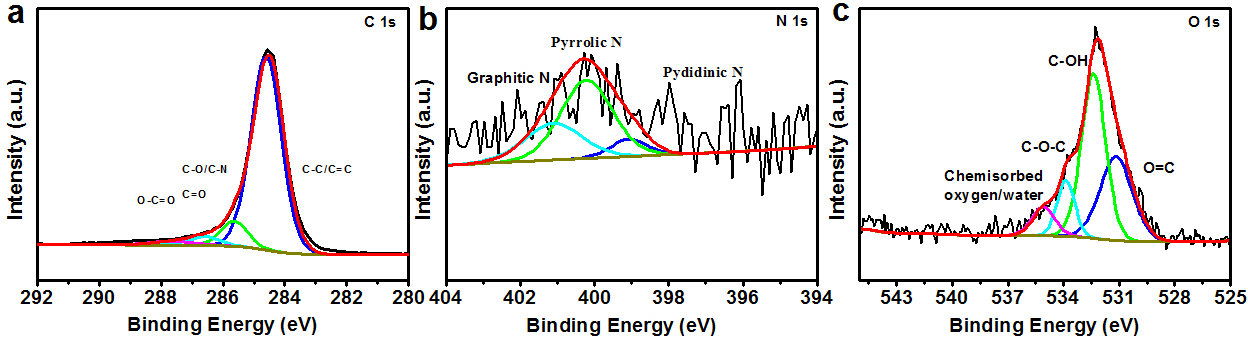


**Fig. S3** (a) C 1s, (b) N 1s, and (c) O 1s XPS spectrum of HTC.


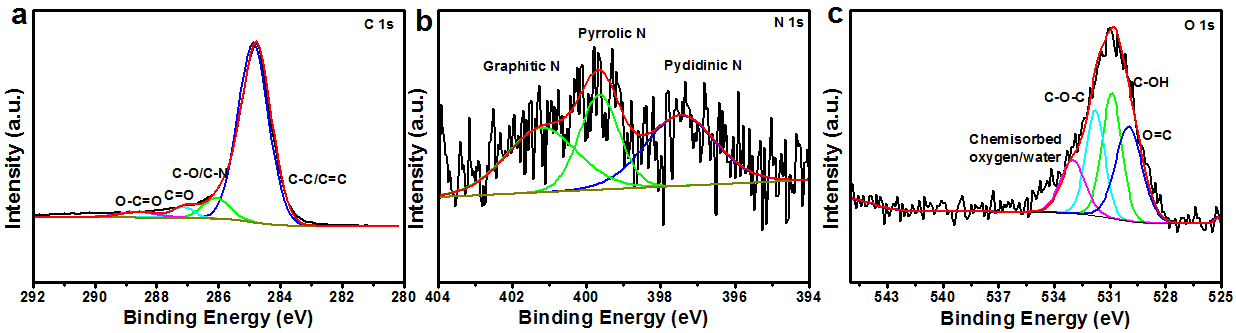


**Fig. S4** (a) C 1s, (b) N 1s, and (c) O1s XPS spectrum of C-KOH.


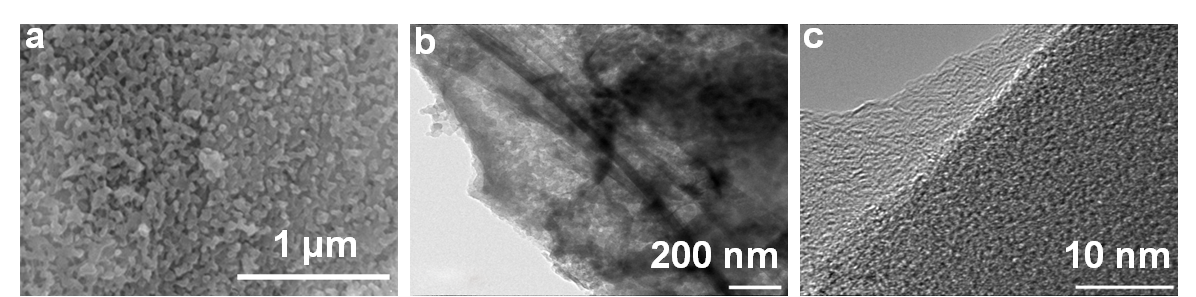


**Fig. S5** (a) SEM, (b) TEM, and (c) HRTEM images of HTC.


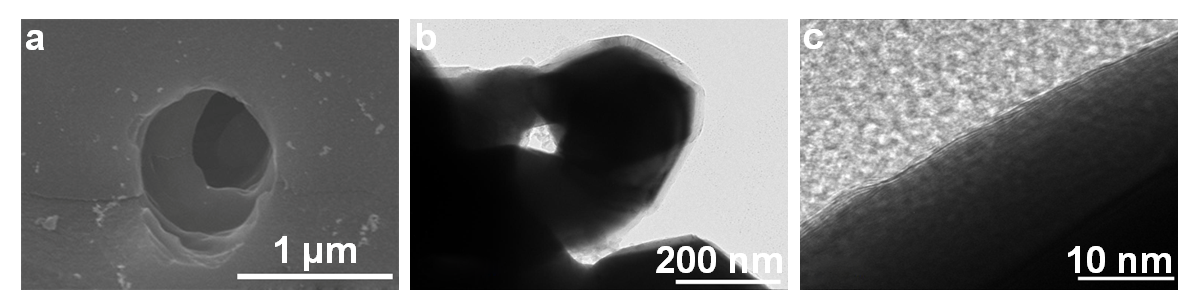


**Fig. S6** (a) SEM, (b) TEM, and (c) HRTEM images of C-KOH.


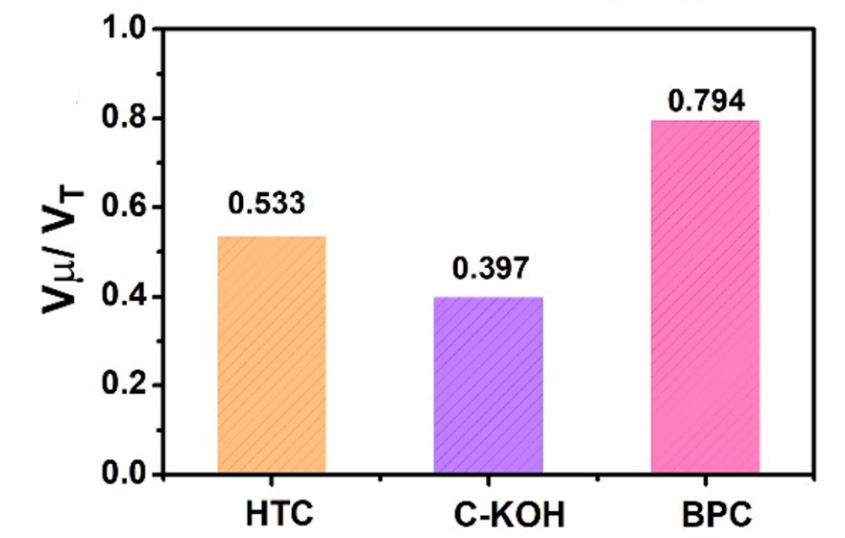


**Fig. S7** Contribution of micropore volume to the total pore volume for HTC, C-KOH, and BPC.


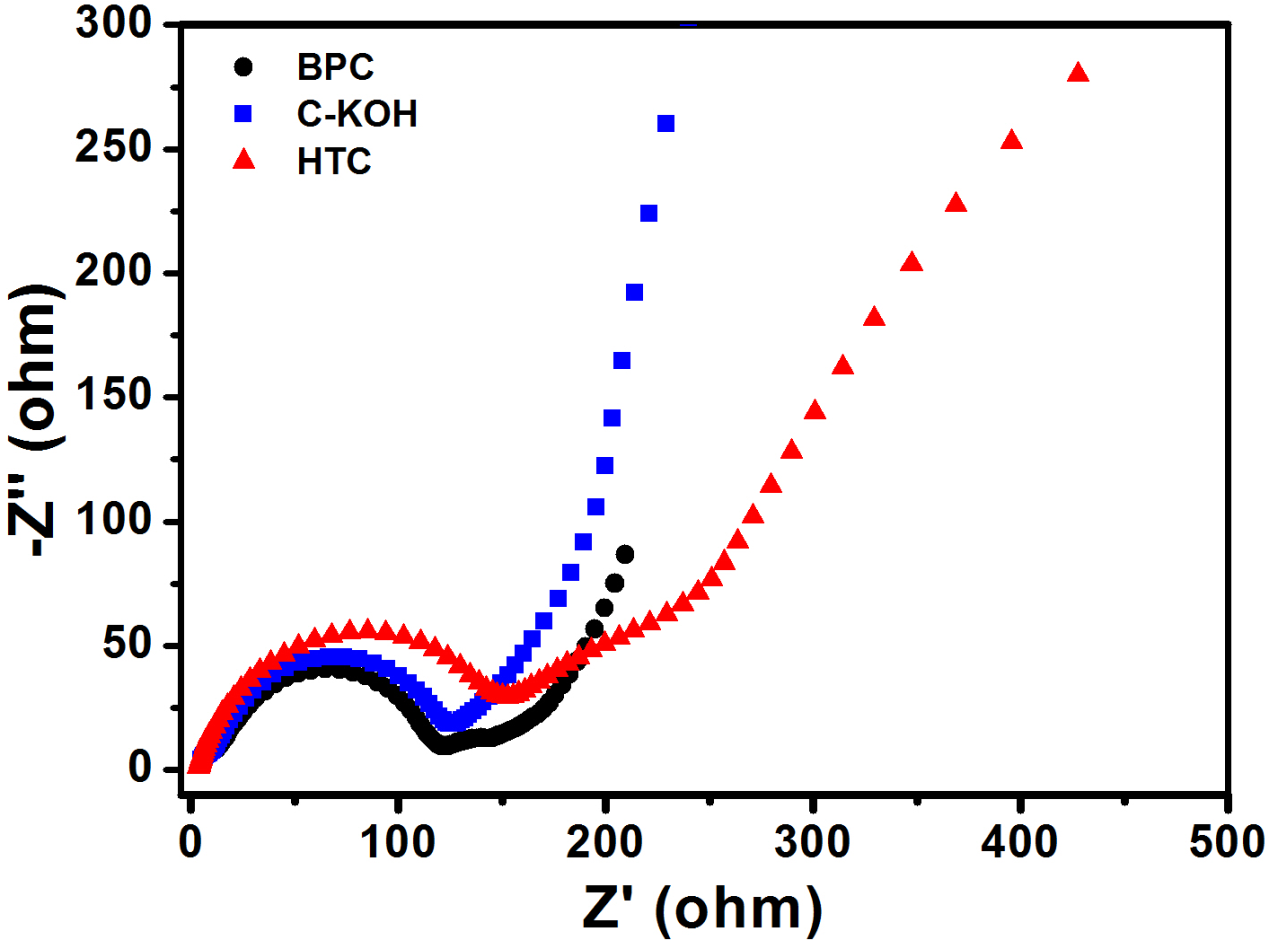


**Fig. S8** Nyquist plots of BPC, HTC, and C-KOH.


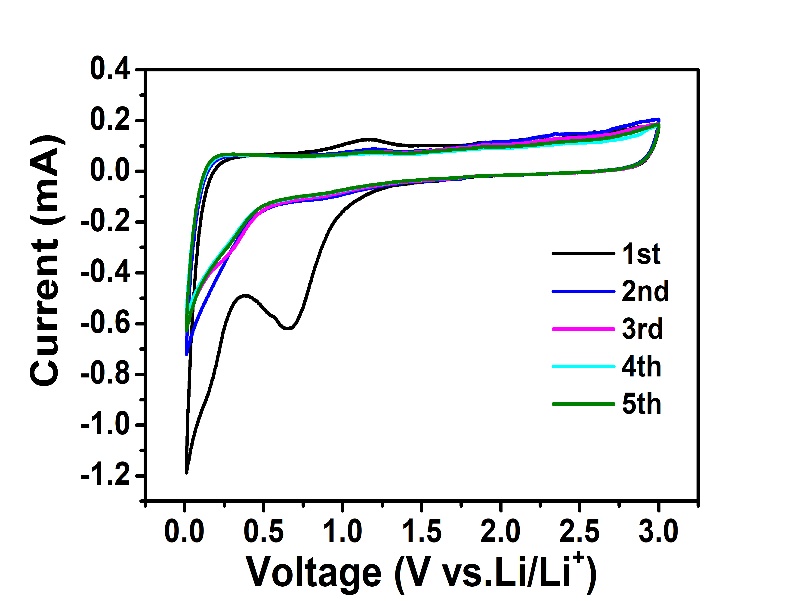


**Fig. S9** CV curves of the BPC sample at a scan rate of 0.1 mV s^-1^.


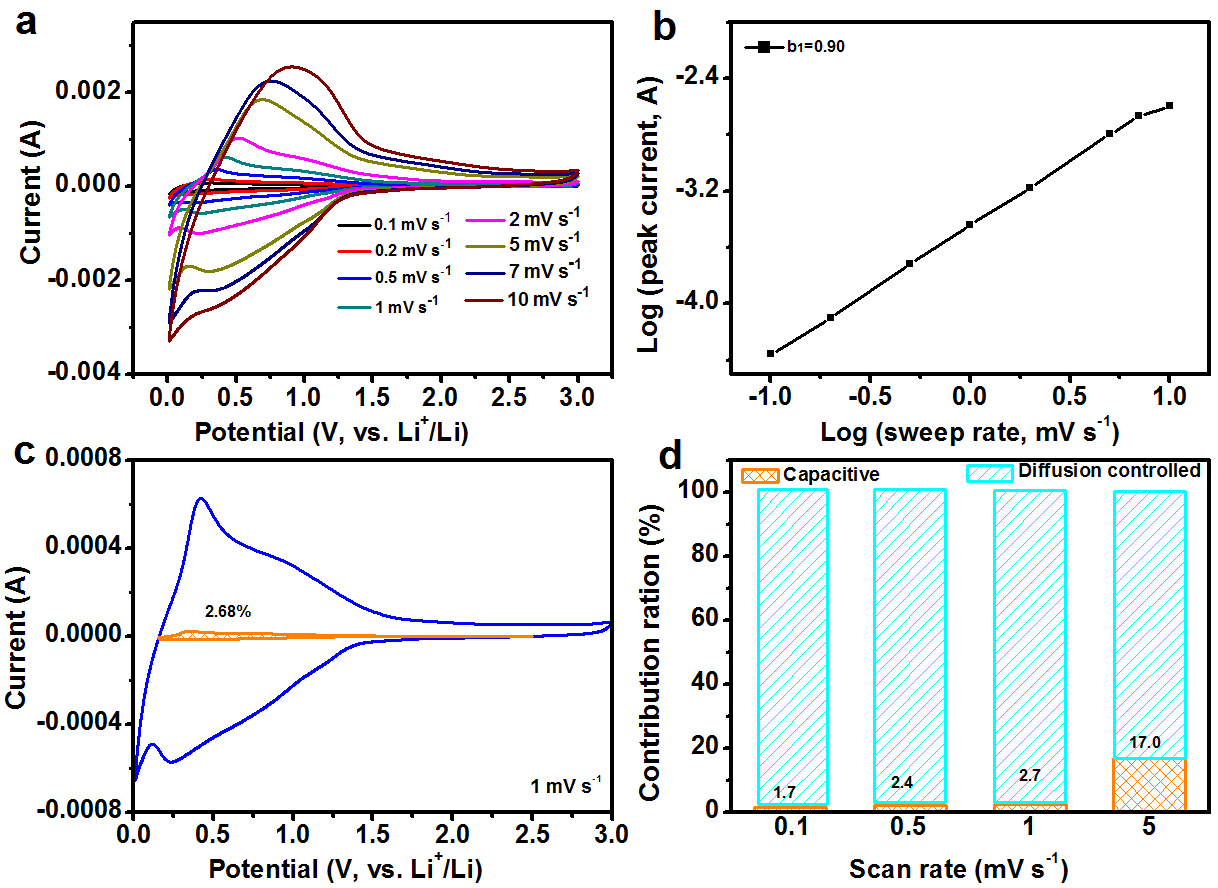


**Fig. S10** (a) CV curves of the HTC electrode at various scan rates from 0.1 to 10 mV s^-1^. (b) Determination of the b value using the relationship between peak current and scan rate. The redox peak at 0.7 V was used for the calculations. (c) Separation of the capacitive and diffusion currents at a scan rate of 1 mV s^-1^. (d) Contribution ratio of the capacitive and diffusion-controlled charge versus scan rate.


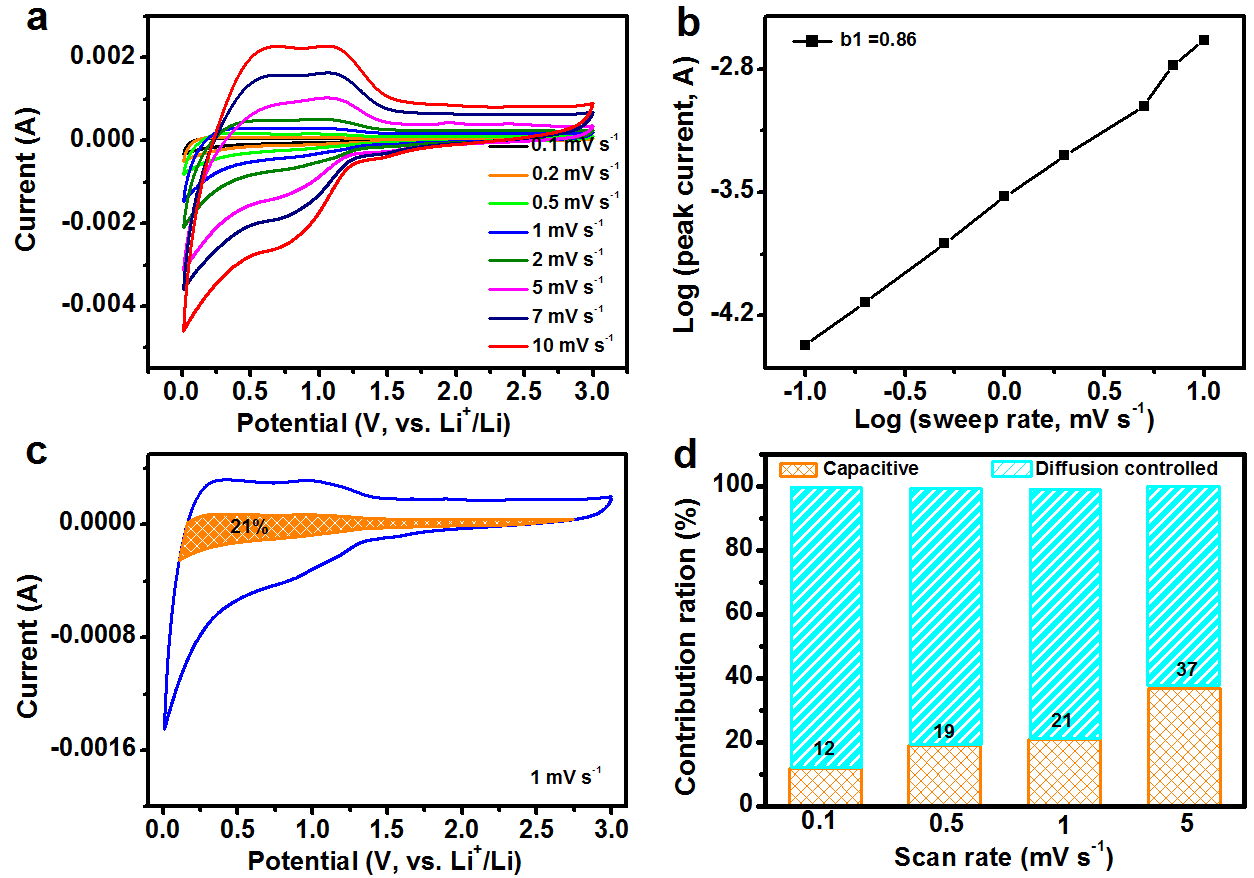


**Fig. S11** (a) CV curves of the C-KOH electrode at various scan rates from 0.1 to 10 mV s^-1^. (b) Determination of the b value using the relationship between peak current and scan rate. The redox peak at 0.7 V was used for the calculations. (c) Separation of the capacitive and diffusion currents at a scan rate of 1 mV s^-1^. (d) Contribution ratio of the capacitive and diffusion-controlled charge versus scan rate.
